# Prevalence of self-reported coronary heart disease and its associated risk factors in Tabari cohort population

**DOI:** 10.1186/s12872-020-01526-w

**Published:** 2020-05-19

**Authors:** Ali Ghaemian, Maryam Nabati, Majid Saeedi, Motahareh Kheradmand, Mahmood Moosazadeh

**Affiliations:** 1 Department of Cardiology, Faculty of Medicine, Cardiovascular Research Center, Mazandaran University of Medical Sciences, Sari, Iran; 2 Department of Pharmaceutics, School of Pharmacy, Mazandaran University of Medical Sciences, Sari, Iran; 3 Health Science Research Center, Addiction Institute, Mazandaran University of Medical Sciences, Sari, Iran

**Keywords:** Cardiovascular disease, Low and middle-income countries, Risk factors, Prevalence

## Abstract

**Background:**

Prevalence of coronary heart disease (CHD) risk factors are increasing in developing countries. The present study aimed to assess the prevalence of self-reported CHD and evaluate the role of various risk factors on its prevalence in the Tabari cohort study (TCS) population.

**Methods:**

The enrollment phase of TCS was performed between June 2015 and November 2017. In the current study, data were derived from information collecting from the enrollment phase of TCS. In the enrollment phase, 10,255 individuals aged 35–70 living in urban and mountainous areas of Sari (northern part of Iran) were entered into the study. Educational level, socioeconomic and marital status, history of smoking, opium and alcohol abuse/addiction, level of daily physical activity, indices of obesity, and traditional risk factors of the participants were determined.

**Results:**

The prevalence of CHD was measured at 9.2%. Older individuals (*P*<0.001), people with a body mass index≥30kg/m2 (*P*<0.001), diabetics (*P*<0.001), and hypertensive (*P*<0.001) have been shown to have an increased risk for CHD compared with participants of without CHD. Furthermore, the CHD was more prevalent in individuals with higher waist circumference (*P*<0.001), higher low-density lipoprotein cholesterol (*P*<0.001), lower high-density lipoprotein cholesterol (*P*<0.001), and a higher waist to hip ratio (*P*<0.001). In addition, individuals with low socioeconomic status, illiterate people, and opium users had a higher prevalence of CHD (*P*<0.001). The results of the multivariable logistic regression analysis showed that the probability of CHD among individuals who had 8-10 risk factors was estimated at 8.41 (95% confidence interval: 5.75-12.31) times higher than those with less than 3 risk factors.

**Conclusion:**

According to the results of the present study, it seems that the prevalence of CHD in the Iranian population is relatively high.

## Background

Cardiovascular disease is the most common cause of mortality in the world and its prevalence is increasing in the developing countries [1]. In recent years, age-adjusted mortality rates of CHD in high-income countries have declined significantly in spite of the increased burden of CHD [2]. Furthermore, the majority of related deaths in these countries occur in retirement ages [3]. This decline in CHD mortality is closely related to both primary and secondary preventive strategies and sustaining health promotion programs [4]. However, in the middle- and low-income countries, CHD remains the main cause of death and the greatest source of disease burden. In these countries, a large proportion of related deaths occur during productive and active ages [5]. Several factors, such as genetic inheritance, social, environmental, and lifestyle-related physical activity can affect the CHD risk. Some of these risk factors, including cigarette smoking, unhealthy diet, physical inactivity, and alcohol use are modifiable [6]. On the other hand, prevalence of traditional risk factors for CHD, including diabetes mellitus (DM), cigarette smoking, physical inactivity, overweightness and obesity, hypertension (HTN), and hyperlipidemia (HLP) are increasing in developing countries [7]. In the present study, data from the Tabari cohort were analyzed to determine the prevalence of self-reported CHD and evaluate the role of different anthropometric, behavioral, and traditional risk factors on the prevalence of self-reported CHD in northern part of Iran.

## Methods

In the current study, data were derived from information collected in the enrollment phase of TCS population, which is a part of national cohort studies named the Prospective Epidemiological Research Studies in Iran (PERSIAN). The participants of TCS have been selected from areas of northern part of Iran (Sari city, Mazandaran province). Inclusion criteria included persons in the age group of 35-70 years, persons only officially resident and Iranian nationality. Exclusion criteria of the study included persons who did not unwillingness to participate in TCS for any reason, and persons who were not able to communicate, inability to speak, deafness and having acute mental disorders. The enrollment phase of TCS was performed between June 2015 and November 2017. We have enrolled 10255 participants aged 35-70 years in TCS. Among them 68.4% lived in urban area and 40.5% of them were men. The questionnaire used for the data collection was a standardized questionnaire which has been explained in detail in methodology and cohort profiling studies [8, 9]. Data regarding educational level, socioeconomic and marital status, history of cigarette or hookah smoking, drug and alcohol abuse/addiction, and level of daily physical activity were collected by a face to face interview. The absolute physical activity intensities were expressed in metabolic equivalents (METs). One MET was defined as 1 kcal/kg/h. Based on calculated METs, the intensity of physical activity was divided into four levels within 24 h: sedentary, low, medium, and high activities. Socioeconomic status was broken into five levels on the basis of 13 variables [6, 8, 9]. Anthropometric measurements were determined by trained personnel, in accordance with standard protocol [10]. According to WHO classification, individuals were categorized into two groups: with a healthy WHR (0.9 or less and 0.85 or less in men and women, respectively), and with a high-risk WHR (0.91 or higher in men and 0.86 or higher in women). The waist to height ratio (WHtR) was calculated by dividing waist circumference by height. Patients were then categorized into two groups: non-obese with a WHtR<0.5 and obese patients with a WHtR>0.5 [11]. Also, blood pressure and laboratory data such as fasting blood glucose (FBS), total cholesterol and, HDL cholesterol were measured in TCS. The HTN was defined as having a systolic blood pressure ≥140 mmHg, diastolic blood pressure ≥90 mmHg, or requiring antihypertensive medications [12]. The DM was defined according to the guidelines of the American Diabetes Association (FBS ≥126mg/dl) or consuming oral hypoglycemic agents or insulin [13]. The HLP was characterized by a serum total cholesterol (TC) level <200mg/dl, high-density lipoprotein-cholesterol (HDL-C) level less than 40mg/dl in men, or less than 50mg/dl in women [14]. Based on the literature, patients with a low-density lipoprotein-cholesterol (LDL-C) level ≥130 mg/dl are at risk for future adverse cardiovascular events [15]. Therefore, we divided the patients into two groups according to serum LDL-C: a group with LDL-C levels<130 mg/dl and those with levels≥130 mg/dl. Hypertriglyceridemia was defined as a plasma triglyceride (TG) level of ≥150 mg/dl [16].

In Tabari cohort, individuals were considered to have CHD if they had one or more of the following factors: history of stable angina pectoris, unstable angina, prior myocardial infarction, heart failure, percutaneous coronary intervention or coronary artery bypass graft surgery. Two trained nurses were in charge of obtaining detailed medical history including CHD from each participant. The question was “have you ever been diagnosed for CHD by a physician?”. If the answer was "Yes", all the medical records of the patients were reviewed by the physicians. Ten variables were regarded as risk factors for CHD. These include: TG≥150mg/dl, TC≥200mg/dl, LDL-C≥130mg/dl, and HDL-C<50mg/dl in women and <40mg/dl in men, BMI≥30kg/m2, less than moderate physical activity, WHR>0.9 for men and >0.85 for women, WHtR≥0.5, DM, and HTN. It should be noted, family history of CHD in first and second degree of relatives was available, but it was not included in the analyses due to possible dilution of the association.

### Statistical analysis

Continuous variables were expressed by mean and categorical variables by frequency and percentile. Patients were categorized into two groups based on having or not having self-reported CHD. An independent t-test was used to compare the means between the two groups. Categorical variables were compared by Chi-square. Furthermore, a multivariable logistic regression analysis was used to adjust the confounding effects of other variables on the prevalence of CHD. A *p*-value of less than 0.05 was considered statistically significant. We used SPSS 16 for analyzing the data of study (SPSS Inc., Chicago, Illinois, USA).

## Results

**Fig. 1:**
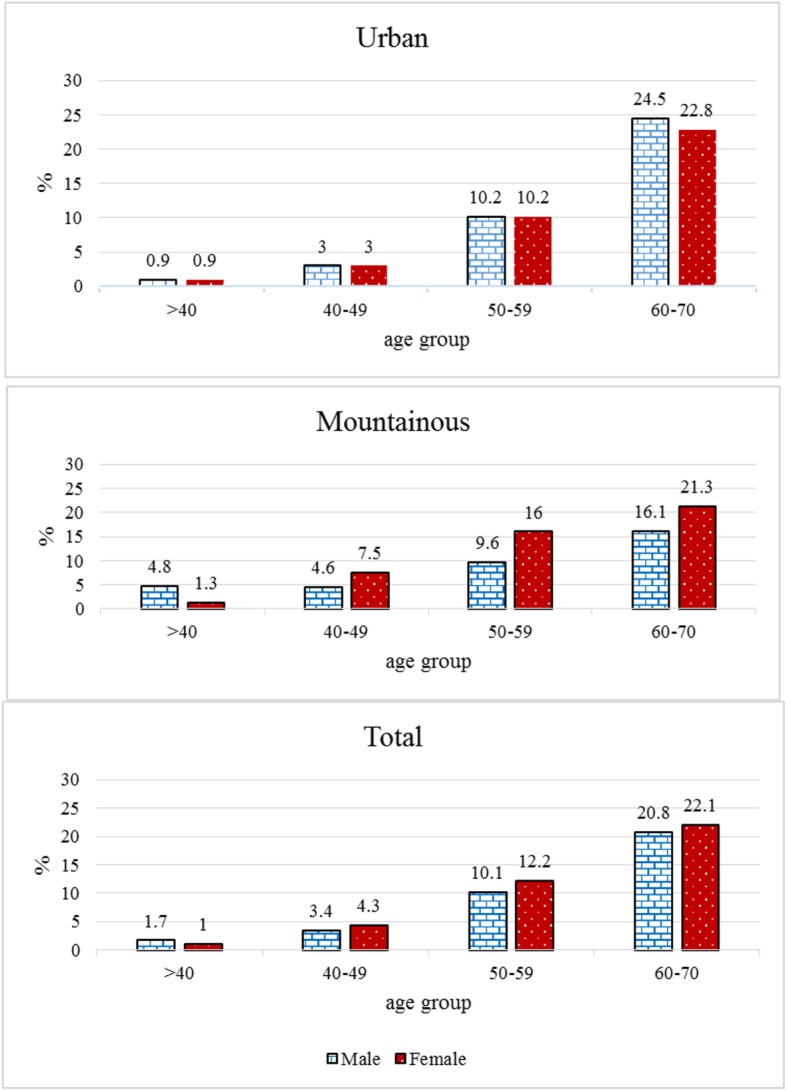
Prevalenc of CHD categorized by age groups (years), sex and area of residence based on data of the enrollment phase of Tabari cohort study from Iran

According to the results of the enrollment phase of TCS, prevalence of CHD was calculated at 9.2% (946/10,255 individuals, confidence interval (CI) 95%:8.7-9.8). The mean age of CHD patients was 51.24±9.45 years. Disease onset was at the age of less than 46, between 46-57, and more than 57 years in 25%, 50%, and 25% of CHD patients, respectively. The age of the disease onset showed no significant difference between men and women (51.78±9.56 vs. 50.87±9.36, *P*=0.146). As it is presented in Fig. 1, in urban population the prevalence of self-reported CHD did not show any difference between men and women. Among the population living in rural regions, the prevalence of CHD was higher in men than women (4.8% vs. 1.3%, respectively) and in participants younger than 40 years. However, its prevalence was higher in women than men in other age groups.

The prevalence of different demographic and anthropometric variables are presented in Table 1. There was no difference in the prevalence of cardiovascular disease between men and women (*P*=0.959). However, the CHD prevalence increased with age (*P*<0.001). Prevalence of CHD in individuals with a BMI≥30kg/m2 was higher than in those with a BMI<25 kg/m2 (11.2% vs. 7.3%, *P*<0.001), in diabetics higher than in non-diabetics (18.1% vs. 7.4%, *P*<0.001), and in hypertensives higher than normotensives (20.9% vs. 5.9%, *P*<0.001). Furthermore, the CHD was more prevalent in individuals with a higher WC (11.0% vs. 7.5%, *P*<0.001), lower HDL-C (10.7% vs. 8.5%, *P*<0.001), higher LDL-C (9.8% vs. 5.8%, *P*<0.001), higher TC (13.5% vs. 5.6%, *P*<0.001), and a higher WHR (11.5 vs. 5.5%, *P*<0.001). In addition, individuals with low socioeconomic status, illiterate people, and opium users had a higher prevalence of CHD (*P*<0.001).

**Table 1:** Demographic and anthropometric variables of the study population categorized by having or not having self-reported CHD in men and women aged 35-70 years of Tabari cohort study enrolment phase from Iran

Variables		*N*=946	*N*=9309	*P*-value (chi square test)
		With CHD; n (%)	Without CHD; n (%)	
Gender	Male	382(9.2)	3767(90.8)	0.959
	Female	564(9.2)	5542(90.8)	
Age (years)	<40	20(1.2)	1592(98.8)	<0.001
	40-49	135(3.9)	3296(96.1)	
	50-59	365(11.3)	2865(88.7)	
	60-70	426(21.5)	1556(78.5)	
Residency	Urban	555(7.9)	6457(92.1)	<0.001
	Rural	391(12.1)	2852(87.9)	
Education level	University/college	155(6.5)	2219(93.5)	<0.001
	9-12 years education	170(5.9)	2726(94.1)	
	6-8 years education	103(9.2)	1018(90.8)	
	1-5 years education	256(11.0)	2076(89.0)	
	illiterate	262(17.1)	1270(82.9)	
Social economic	1	246(12.0)	1805(88.0)	<0.001
	2	223(10.9)	1829(89.1)	
	3	184(9.0)	1866(91.0)	
	4	140(6.8)	1911(93.2)	
	5	153(7.5)	1898(92.5)	
Marital state	Married	863(9.2)	8557(90.8)	0.456
	Single-widow-divorce	83(9.9)	752(90.1)	
BMI (kg/m^2^)	<25	181(7.3)	2292(92.7)	<0.001
	25-29.9	381(8.8)	3962(91.2)	
	≥30	384(11.2)	3055(88.8)	
Cigarette smoking	No	876(9.4)	8450(90.6)	0.062
	Yes	70(7.5)	859(92.5)	
Alcohol user	No	876(9.3)	8573(90.7)	0.581
	Yes	70(8.7)	736(91.3)	
Opium user	No	861(8.9)	8770(91.1)	<0.001
	Yes	85(13.6)	539(86.4)	
Hookah user	No	906(9.4)	8766(90.6)	0.042
	Yes	40(6.9)	543(93.1)	
Physical activity	Sedentary	282(11.0)	2276(89.0)	0.003
	Mild intensity	212(8.2)	2360(91.8)	
	Moderate intensity	221(8.6)	2341(91.4)	
	Severe intensity	231(9.0)	2332(91.0)	
Diabetic	No	626(7.4)	7864(92.6)	<0.001
	Yes	320(18.1)	1445(81.9)	
Hypertensive	No	470(5.9)	7504(94.1)	<0.001
	Yes	476(20.9)	1805(79.1)	
WC (cm)	men<102 and women<88	392(7.5)	4837(92.5)	<0.001
	men≥102 and women ≥88	554(11.0)	4472(89.0)	
WHtR	<0.50	67(4.8)	1326(95.2)	<0.001
	>=0.50	879(9.9)	7983(90.1%)	
HDL-C (mg/dl)	men≥40 and women ≥50	575(8.5)	6225(91.5)	<0.001
	men<40 and women<50	371(10.7)	3084(89.3)	
LDL-C (mg/dl)	<130	83(5.8)	1357(94.2)	<0.001
	≥130	863 (9.8)	7952(90.2)	
TC (mg/dl)	<200	310(5.6)	5221(94.4)	<0.001
	≥200	636(13.5)	4088(86.5)	
TG (mg/dl)	<150	286(5.3)	5069(94.7)	<0.001
	≥150	660(13.5)	4240(86.5)	
WHR	men≤0.9 and women ≤0.85	177(5.5)	3068(94.5)	<0.001
	men>0.9 and women >0.85	769(11.0)	6241(89.0)	
Number of risk factors	None	1(1.2)	80(98.8)	<0.001
	one	13(3.2)	399(96.8)	
	two	23(3)	744(97)	
	Three	59(5.1)	1106(94.9)	
	four	78(4.7)	1592(95.3)	
	five	128(6.2)	1936(93.8)	
	Six	204(10.7)	1711(89.3)	
	Seven	205(16)	1077(84)	
	Eight -Ten	235(26.1)	664(73.9)	

*CHD* Coronary heart disease, *WC* Waist circumference, *WHR* Waist to hip ratio, *WHtR* Waist to height ratio, *BMI* Body mass index, *HDL* High-density lipoprotein cholesterol, *LDL* Low-density lipoprotein cholesterol, *TC* Total cholesterol, *TG* Triglyceride

Multivariable logistic regression analysis showed that among different variables, older individuals aged 60-70 years had 10.51 times greater risk of having CHD than those aged 35-40 years, rural population 1.69 times than urban population, opium users 1.72 times than non-drug users, diabetics 1.28 times than non-diabetics, hypertensives 2.23 times than normotensives and individuals with a low HDL-C 1.51 times than those with high HDL-C. Moreover, individuals with intense physical activity were 37% less at risk of having CHD than those with a sedentary lifestyle (Table 2).

**Table 2:** Independent demographic and anthropometric Predictors of CHD according to multivariable logistic regression analysis in men and women aged 35-70 years of Tabari cohort study enrolment phase from Iran

Variable		OR	CI 95%	*P*-value
Gender	Male	1	1	1
	Female	0.83	0.67-1.02	0.077
Age (years)	<40	-	-	-
	40-49	2.47	1.53-4.00	<0.001
	50-59	5.86	3.67-9.37	<0.001
	60-70	10.51	6.51-16.97	<0.001
Residency	Urban	1	1	1
	Rural	1.69	1.36-2.10	<0.001
Education level	University/college	1	1	1
	9-12	0.83	0.65-1.06	0.127
	6-8	1.14	0.84-1.53	0.403
	1-5	1.18	0.89-1.56	0.230
	illiterate	1.09	0.79-1.50	0.613
Social economic	1	1	1	1
	2	1.07	0.86-1.33	0.547
	3	1.16	0.89-1.50	0.256
	4	1.08	0.81-1.45	0.596
	5	1.01	0.74-1.38	0.934
Marital state	Single-widow-divorce	1	1	1
	Married	1.20	0.92-1.57	0.177
BMI (kg/m^2^)	<25	1	1	1
	25-29.9	0.93	0.73-1.19	0.568
	≥30	1.07	0.81-1.42	0.637
Cigarette smoking	No	1	1	1
	Yes	0.88	0.64-1.19	0.400
Alcohol user	No	1	1	1
	Yes	1.04	0.76-1.42	0.796
Opium user	No	1	1	1
	Yes	1.72	1.28-2.31	<0.001
Hookah user	No	1	1	1
	Yes	0.83	0.58-1.20	0.327
Physical activity	sedentary	1	1	1
	Mild intensity	0.85	0.69-1.05	0.134
	Moderate intensity	0.81	0.66-1.00	0.050
	Severe intensity	0.63	0.49-0.80	<0.001
Diabetic	No	1	1	1
	Yes	1.28	1.08-1.51	0.003
Hypertensive	No	1	1	1
	Yes	2.23	1.92-2.60	<0.001
WC (cm)	men<102 and women<88	1	1	1
	men≥102 and women ≥88	0.98	0.78-1.23	0.852
HDL-C (mg/dl)	men≥40 and women ≥50	1	1	1
	men<40 and women<50	1.51	1.29-1.77	<0.001
LDL-C (mg/dl)	<130	1	1	1
	≥130	0.60	0.45-0.80	0.001
TC (mg/dl)	<200	1	1	1
	≥200	1.89	1.57-2.27	<0.001
TG (mg/dl)	<150	1	1	1
	≥150	1.81	1.50-2.17	<0.001
WHR	men≤0.9 and women ≤0.85	1	1	1
	men>0.9 and women >0.85	0.96	0.77-1.20	0.740
WHtR	<0.5	1	1	1
	>=0.5	1.36	0.96-1.93	0.085

*CHD* Coronary heart disease, *WC* Waist circumference, *WHR* Waist to hip ratio, *WHtR* Waist to height ratio, *BMI* Body mass index, *HDL* High-density lipoprotein cholesterol, *LDL* Low-density lipoprotein cholesterol, *TC* Total cholesterol, *TG* Triglyceride

Table 3 represents the risk of developing CHD based on the number of individual's risk factors to adjust the effects of probable confounding variables which has been set according to the analysis from a hierarchical model. Model 1 evaluates the risk of developing CHD without adjusting the effects of other variables (crud model); model 2 describes the adjusted effect of gender; model 3 manifests the adjusted effect of age. In addition, model 4 demonstrates adjusted effects of socioeconomic status, level of education, marital status, area of residency, hookah or drug abuse, cigarette smoking, and alcohol consumption and model 5 shows adjusted effects of all included variables in models 2-4. All five models showed that the chance of having CHD increased significantly with increasing number of risk factors. Based on full model results (model 5), chance of having CHD in subjects with 3, 4, 5, 6, 7, and 8-10 risk factors were 1.94, 1.62, 2.02, 3.37, 4.91, and 8.41 times higher (95%CI), respectively, compared to those with less than 3 risk factors.

**Table 3:** The relationship between various risk factors and CHD by number of risk factors according to multivariable logistic regression analysis in men and women aged 35-70 years of Tabari cohort study enrolment phase from Iran

Model	Number of risk factors	OR	CI 95%	*P*-value
Model 1 (Crud)	0-2	1	1	1
	3	1.76	1.16-2.68	0.008
	4	1.62	1.09-2.41	0.018
	5	2.18	1.50-3.17	<0.001
	6	3.94	2.76-5.64	<0.001
	7	6.29	4.39-9.01	<0.001
	8-10	11.69	8.17-16.76	<0.001
Model 2 (Adjusted based on Gender)	0-2	1	1	1
	3	1.82	1.20-2.77	0.005
	4	1.69	1.13-2.52	0.010
	5	2.32	1.60-3.37	<0.001
	6	4.24	2.96-6.07	<0.001
	7	6.90	4.81-9.92	<0.001
	8-10	13.34	9.27-19.22	<0.001
Model 3 (Adjusted based on age)	0-2	1	1	1
	3	1.78	1.16-2.72	0.008
	4	1.46	0.97-2.19	0.066
	5	1.78	1.22-2.59	0.003
	6	2.92	2.03-4.20	<0.001
	7	4.21	2.92-6.08	<0.001
	8-10	7.18	4.97-10.36	<0.001
Model 4 (Adjusted based on social economic, education level, area of residence, marital status, cigarette smoking, hookah smoking, opium addiction, use of alcohol)	0-2	1	1	1
	3	1.84	1.20-2.79	0.005
	4	1.70	1.14-2.53	0.010
	5	2.20	1.51-3.21	<0.001
	6	3.89	2.70-5.59	<0.001
	7	5.95	4.12-8.58	<0.001
	8-10	10.59	7.33-15.30	<0.001
Model 5 (Adjusted based on age, gender, social economic, education level, area of residence, marital status, cigarette smoking, hookah smoking, opium addiction, use of alcohol)	0-2	1	1	1
	3	1.94	1.26-2.98	0.003
	4	1.62	1.08-2.44	0.021
	5	2.02	1.37-2.97	<0.001
	6	3.37	2.32-4.89	<0.001
	7	4.91	3.37-7.16	<0.001
	8-10	8.41	5.75-12.31	<0.001

## Discussion

According to the findings of the present study, the prevalence of self-reported CHD was measured at 9.2%. We found no difference between the age of onset and prevalence of CHD between men and women. The CHD was more prevalent in older patients, people with a BMI ≥ 30kg/m2, diabetic, hypertensive, and individuals with a higher WC, WHR, LDL-C, TC, and a lower HDL-C. In addition, rural populations, individuals of lower socioeconomic status, illiterate people, and opium users had higher prevalence of CHD. Moreover, a greater number of CHD risk factors were associated with higher prevalence of self-reported CHD.

CHD is the most common cause of mortality in Iran. Prevalence of cardiovascular risk factors in Iran is increasing and almost half of all deaths per year and a high proportion (79%) of deaths related to chronic illnesses are caused by CHD. Also, a large proportion of CHD-related deaths occur during reproductive age. The impact of these events on health makes them as the most important health related problems in this country [5]. The prevalence of self-reported angina pectoris among Canadians aged 12 years old or older was reported about 5% and in the US adults aged 20 years or older was 6.9% [14]. More than 75% of all CHD-related deaths occur in developing countries. In India, the prevalence of CHD is 9-10% among urban and 4-6% in rural populations [15]. The prevalence of self-reported CHD in the current study was calculated at 9.2%, which was relatively higher than other studies. This heterogeneity may be due to a complex interaction of various genetic and environmental factors that play an important role in the development of atherosclerosis. The prevalence of cardiometabolic risk factors (e.g., central obesity, hyperglycemia, HLP, and HTN) has been reported to be rising in recent years in Iran [17]. Moreover, in the present study only individuals aged 35 years or older were selected and younger people with a low probability of CHD were excluded from the study. In the current research, 75% of CHD patients reported that they developed this disease before the age of 57, which is representative of a relatively early age of disease onset. Previous studies considered the age limit within 35-55 years for defining young coronary artery disease (CAD). The mean age of onset of CAD in Southeast Asians has been reported to be 53 years as compared to European age of 63 years. The incidence of CHD in younger persons is increasing over recent decades in developing countries [18]. This is consistent with findings of the present study.

Alizadeh Sani et al. used a data mining based approach for CAD diagnosis by arranging the features in four categories: demographic, symptoms and physical examination, electrocardiogram, and laboratory and echocardiographic findings. They found that the most important indicators for the detection of CAD patients were typical chest pain, age, HTN, DM, pulse rate, and BMI [19]. Aging, HTN, DM, physical inactivity, overweightness and obesity, and HLP are known as traditional risk factors for the development of atherosclerosis. The probability of developing CHD significantly rises if one or more of these risk factors exist [7]. BMI is an index of obesity that is associated with an increased risk of cardiovascular disease. However, recent studies have shown that the pattern of distribution of body fat is a more important determinant of risk than BMI [20]. Indeed, indices of abdominal obesity including WC, WHR, and WHtR have been linked with higher cardiometabolic risk [21]. In the present study, CHD was more prevalent among hypertensives, diabetics, and individuals with a lower HDL-C and a higher LDL-C, TC, BMI, WC, and WHR.

Furthermore, it was more prevalent among illiterate and poor individuals than people of higher socioeconomic status. Lack of adequate access to health and social facilities causes people to become sicker and die earlier than people with a higher socioeconomic status. Awareness and education about risk factors of daily living activities may be partially responsible for a decrease in the CHD prevalence among individuals whit a high socioeconomic status [22]. Higher education is associated with better lifestyles and preventive measures, lower prevalence of risk factors, early detection and treatment of risk factors, better quality of acute coronary syndrome treatment, and better long-term management and compliance, compared to low education level [23].

In the present study, CHD was more prevalent in rural people than urban individuals. The altered epidemic pattern in rural regions, increased prevalence of cardiovascular risk factors, and an increase in the number of elderly people might explain these differences [24].

The prevalence of opium addiction has risen by three folds for the past 20 years in Iran and it is estimated that 2-2.8% of the population is addicted to opium which may be regarded as a risk factor for CHD. Opioids affect the hypothalamus by inhibiting the release of gonadotropin-releasing hormone, leading to reduction in the concentrations of plasma testosterone. There is a significant inverse correlation between plasma testosterone levels and the extent of CAD. Men with low testosterone levels might have an increased risk for coronary atherosclerosis. Testosterone may have some direct vasodilating effects on coronary vasculature. Consistent with the results of the present study, CHD was more prevalent in opium-addicted individuals than non-addicted ones [25].

### Limitation

We think that our study has some limitations. First in our study some patients might not have been aware of having CHD. This may lead to an underestimation of CHD prevalence in our population. Furthermore, the population of the present study was selected from individuals within the age range of 35-70 years, who were living in the northern part of Iran. Therefore, the results of the present study cannot be generalized to the entire population of Iran. Finally, underestimation of the rate of opium and alcohol use could be another limitation of the present study. However, due to the close relationship between participants and the researchers we think that most patients answered honestly to this part of the questionnaire. On the other hand, the strength of our study is that it includes a large sample size which is more representative of the general population than smaller studies. We also evaluated the socioeconomic status of the population comprehensively

## Conclusion

It seems that the prevalence of self-reported CHD in the Iranian population is relatively high, compared to other nations. In addition, the incidence of CHD in younger subjects is increasing. The rapid increase of CHD risk factors and sedentary lifestyle are the major determinants of a higher prevalence of CHD in this area. However, detrimental effects of inherited genetic risk factors on cardiovascular system should not be overlooked.

## Data Availability

The datasets used and/or analysed during the current study are available from the corresponding author on reasonable request.

## Abbreviations

CHD: Coronary heart disease
TCS: Tabari cohort study
DM: Diabetes mellitus
FBS: Fasting blood glucose
HTN: Hypertension
HLP: Hyperlipidemia
WC: Waist circumference
WHR: Waist to hip ratio
WHtR: Waist to height ratio
TC: Total cholesterol
HDL-C: High-density lipoprotein-cholesterol
LDL-C: Low-density lipoprotein-cholesterol
TG: Triglyceride
